# Exosomes from mesenchymal stem cells induce the conversion of hepatocytes into progenitor oval cells

**DOI:** 10.1186/s13287-017-0560-z

**Published:** 2017-05-23

**Authors:** Hao-Hsiang Wu, Oscar K. Lee

**Affiliations:** 1 Institute of Biophotonics, National Yang-Ming University, No.155, Sec.2, Linong Street, Taipei, 112 Taiwan; 2 Taipei City Hospital, No.145, Zhengzhou Road, Datong District, Taipei, 10341 Taiwan; 3 Institute of Clinical Medicine, National Yang-Ming University, No.155, Sec.2, Linong Street, Taipei, 112 Taiwan; 4 Stem Cell Research Center, National Yang-Ming University, No.155, Sec.2, Linong Street, Taipei, 112 Taiwan; 5 Departments of Medical Research, Taipei Veterans General Hospital, No. 201, Sec. 2, Shipai Road, Taipei, 112 Taiwan

**Keywords:** Mesenchymal stem cells, Paracrine signaling, Conditioned medium, Exosomes

## Abstract

**Background:**

We previously reported that mesenchymal stem cells (MSCs) possess therapeutic effects in a murine model of carbon tetrachloride-induced acute liver failure. In the study, we observed that the majority of repopulated hepatocytes were of recipient origin and were adjacent to transplanted MSCs; only a low percentage of repopulated hepatocytes were from transplanted MSCs. The findings indicate that MSCs guided the formation of new hepatocytes. Exosomes are important messengers for paracrine signaling delivery. The aim of this study is to investigate the paracrine effects, in particular, the effects of exosomes from MSCs, on hepatocytes.

**Methods:**

Mature hepatocytes were isolated from murine liver by a two-step perfusion method with collagenase digestion. MSCs were obtained from murine bone marrow, and conditioned medium (CM) from MSC culture was then collected. Time-lapse imaging was used for observation of cell morphological change induced by CM on hepatocytes. In addition, expression of markers for hepatic progenitors including oval cells, intrahepatic stem cells, and hepatoblasts were analyzed.

**Results:**

Treatment with the CM promoted the formation of small oval cells from hepatocytes; time-lapse imaging demonstrated the change from epithelial to oval cell morphology at the single hepatocyte level. Additionally, expression of EpCAM and OC2, markers of hepatic oval cells, was upregulated. Also, the number of EpCAM^high^ cells was increased after CM treatment. The EpCAM^high^ small oval cells possessed colony-formation ability; they also expressed cytokeratin 18 and were able to store glycogen upon induction of hepatic differentiation. Furthermore, exosomes from MSC-CM could induce the conversion of mature hepatocytes to EpCAM^high^ small oval cells.

**Conclusions:**

In summary, paracrine signaling through exosomes from MSCs induce the conversion of hepatocytes into hepatic oval cells, a mechanism of action which has not been reported regarding the therapeutic potentials of MSCs in liver regeneration. Exosomes from MSCs may therefore be used to treat liver diseases. Further studies are required for proof of concept of this approach.

**Electronic supplementary material:**

The online version of this article (doi:10.1186/s13287-017-0560-z) contains supplementary material, which is available to authorized users.

## Background

Mesenchymal stem cells (MSCs) possess multilineage differential potential and have been shown to possess therapeutic effects in animal models [1, 2]. In our previous study, MSC transplantation restored liver function in mice with carbon tetrachloride (CCl_4_)-induced acute liver failure [3]. However, only a small percentage of repopulated hepatocytes from donor MSCs were observed. Although evidence in the literature has indicated that MSC-derived hepatocytes exhibit long-term persistence after transplantation, tissue integration of transplanted MSCs is rarely observed [4–6]. In fact, recipient hepatocytes significantly participate in liver regeneration-transplanted MSCs. Recently, MSCs have been shown to release trophic factors to repair injured tissues and organs in a paracrine fashion after transplantation [7], and to inhibit apoptosis of hepatocytes and stimulate liver regeneration [8]. Together, action through paracrine signaling is an important mechanism of action for MSCs to treat liver diseases.

Extracellular vesicles (EVs) are secreted microvesicles with the diameter of less than 1 μm that contain bioactive molecules including lipids, proteins, mRNAs, and miRNAs [9]. By fusing with the cell membrane, EVs can interact with target cells and transfer biological molecules to regulate tissue repair [10]. Exosomes are small extracellular vesicles with the diameter of 30 to 120 nm derived from the budding of membranes of multivesicular bodies [11]. Exosomes from stem cells possess therapeutic effects in preclinical studies [12]. Besides, exosomes from MSCs have recently been shown to accelerate hepatic regeneration and alleviate liver fibrosis [13, 14]. Indeed, exosomes are important messengers of paracrine signaling from MSCs and may participate in liver regeneration.

Over 80% of the mass of the liver is comprised of hepatocytes. When liver is damaged, quiescent hepatocytes re-enter the cell cycle and proliferate to achieve liver regeneration [15]. Liver regeneration is a complex and highly controlled network in response to injury. During liver regeneration, oval cells abundantly emerge and these cells act as progenitor cells. It is generally accepted that oval cells are originated from intrahepatic stem cells during development [16]. Epithelial cell adhesion molecule (EpCAM) is highly expressed in intrahepatic stem cells and hepatoblasts, but is not expressed in mature hepatocytes and therefore has been used as a marker for hepatic oval cells [17, 18]. Dynamic expression of EpCAM is also considered to correlate with hepatic differentiation [19]. A recent report further indicates that hepatocytes can undergo dedifferentiation to hepatic progenitor cells and then replenish hepatocytes by in vivo lineage tracing [20]. During the process, formation of hepatic oval cells is identified. However, it is unclear whether paracrine signaling from MSCs triggers the formation of hepatic oval cells. In the current study, we investigate whether MSCs induce formation of hepatic oval cells through paracrine signaling, in particular, through exosomes.

## Methods

### Isolation murine MSCs and hepatocytes

The use of animals was approved by the Taipei Veterans General Hospital Institutional Animal Care and Use Committee (IACUC 2014-043) prior to the commencement of the experiments. The isolation of murine MSCs from the bone marrow of Balb/c mice was performed with our previously reported method [21]. Murine MSCs were then cultured in low-glucose Dulbecco’s modified Eagle’s medium (LGDMEM; Sigma-Aldrich, St. Louis, MO, USA) supplemented with 10% fetal bovine serum (FBS; Thermo Fisher Scientific, Waltham, MA, USA) and 1% penicillin-streptomycin-glutamine (PSG; Thermo Fisher Scientific).

For isolation of hepatocytes, Balb/c mice were euthanized by intraperitoneal injection of tribromoethanol (240 mg/kg), and the two-stage liver perfusion method was used for isolation of primary hepatocytes as previously described [22, 23]. After perfusion, the liver was cut and transferred to a new dish with culture medium consisting of DMEM/F12 (Sigma-Aldrich) supplemented with 10% FBS and 1% PSG. The gall bladder was then removed. When the liver capsule was disrupted by tweezers, the cells were released into the culture medium. The cell suspension was filtered using a 70-μm strainer, and hepatocytes and nonparenchymal cells were then separated at 50 × *g* for 3 minutes. The hepatocyte pellet was washed twice with the culture medium, and hepatocytes were cultured on type I collagen precoated dishes.

### In vitro differentiation of MSCs

- Osteogenic differentiation
  The cells were treated with osteogenic medium consisting of high-glucose DMEM (Sigma-Aldrich) supplemented with 10 mM β-glycerol phosphate (Sigma-Aldrich), 50 μg/mL ascorbic acid (Sigma-Aldrich), and 100 nM dexamethasone (Sigma-Aldrich) for 2 weeks. Osteogenic differentiation of cells was then assessed by alkaline phosphatase and von Kossa staining.
- Adipogenic differentiation
  The cells were treated with adipogenic medium consisting of high-glucose DMEM supplemented with 10% FBS (SAFC Bioscience, St. Louis, MO, USA), 5 μg/mL insulin (Sigma-Aldrich), 50 μM indomethacin (Sigma-Aldrich), 1 μM dexamethasone, and 0.5 mM 3-isobutyl-1-methylxanthine (IBMX; Sigma-Aldrich) for 2 weeks. Oil red O staining was used to assess adipogenic differentiation.
- Chondrogenic differentiation
  To induce chondrogenesis, 2 × 10^5^ cells were centrifuged at 50 × *g* for 5 minutes, pelleted, and treated with chondrogenic medium, consisting of high-glucose DMEM supplemented with 500 ng/mL BMP-6 (R&D Systems, Minneapolis, MN, USA), 10 ng/mL transforming growth factor (TGF)-beta 3 (R&D Systems), 100 nM dexamethasone, 50 μg/mL ascorbic acid, 40 μg/mL proline (Sigma-Aldrich), 100 μg/mL pyruvate (Sigma-Aldrich), and 50 mg/mL ITS^+^ premix (6.25 μg/mL insulin, 6.25 μg/mL transferrin, 6.25 ng/mL selenious acid, 1.25 mg/mL bovine serum albumin [BSA], and 5.35 mg/mL linoleic acid; BD Biosciences, Franklin Lakes, NJ, USA). Chondrogenic differentiation was assessed by Safranin O staining.

### Preparation of CM

MSCs were cultured in 100-mm dishes until optimal confluency. After aspirating the culture medium, the MSCs were treated with 8 ml LGDMEM supplemented with 0.5% FBS and 1% PSG for 3 days, and then the MSC-CM was collected. The cell debris was removed using 0.22-μm filters. Protein concentration was determined for every batch of collected MSC-CM. Fresh preparation of LGDMEM supplemented with 0.5% FBS and 1% PSG was also prepared as control medium.

### Preparation of exosomes

MSC-CM was concentrated by a 3KDa Vivaspin concentrator (GE Healthcare, Chicago, IL, USA), and then Exoprep (Hansa BioMed, Tallinn, Estonia) was used for isolation of exosomes. The exosomes were also identified using western blotting, dynamics light scattering (HORIBA SZ-100, Kyoto, Japan) analysis and transmission electronic microscopy (TEM) (HITACHI HT7700, Tokyo, Japan) was performed.

### Flow cytometry

For flow cytometry analysis, 2 × 10^5^ cells were washed with phosphate-buffered saline (PBS) and then stained with the following antibodies: anti-CD11b (BD Pharmingen, Franklin Lakes, NJ, USA), anti-CD29 (eBioscience, San Diego, CA, USA), anti-CD31 (BD Pharmingen), anti-CD34 (BD Pharmingen), anti-CD44 (BD Pharmingen), anti-CD45 (BD Pharmingen), anti-CD73 (eBioscience), anti-CD105 (eBioscience), anti-CD90 (BD Pharmingen), anti-CD117 (BD Pharmingen), anti-Sca1 (BD Pharmingen), anti-CD26 (eBioscience), anti-EpCAM (Abcam), and goat anti-rabbit IgG PE-Cy5.5 (Thermo Fisher Scientific).

### Quantitative polymerase chain reaction (PCR)

The RNA was collected and reverse-transcribed to cDNA by MMLV reverse transcriptase (EPICENTRE Biotechnologies, Madison, WI, USA). The primer sequences are described in Table 1. The total volume for quantitative PCR was 10 μL, containing 10 ng template cDNA in 4 μL of nuclease-free water, 5 μL 2 × SYBR Green I Master Mix (Thermo Fisher Scientific), and 0.5 μL of 10 μM forward and reverse primers.

**Table 1:** Primer list

Target	Sequence
Alpha fetoprotein	Forward: CACACCCGCTTCCCTCAT Reverse: CAAACTCATTTTCGTGCAATGC
Cytokeratin 19	Forward: GACCTGCGTCCCTTTTTCCT Reverse: CTGAGGTCTGGCGATAGCTATAGG
γ-glutamyltranspeptidase (GGT)	Forward: TCACAGCCCAGATTGTGAAA Reverse: TCAGCTCAGCACGGTAGTTG
Thy1	Forward: ATCCCCCAGACAGCGAGAGT Reverse: CGCCTGCCCCTGAGATT
CD34	Forward: GGGTAGCTCTCTGCCTGATG Reverse: TCCGTGGTAGCAGAAGTCAA
Lgr5	Forward: GACTTTAACTGGAGCAAAGATCTC Reverse: CGAGTAGGTTGTAAGACAAATCTA
DLK1	Forward: GTAGCAAAAGCGGGAAAGC Reverse: GGGTCCTAACTTCAGCCACA
Onecut 2 (OC2)	Forward: CCGAGTTCCAGCGCATGT Reverse: TCTTTGTTTGGTTCTTGCTCTTTG
EpCAM	Forward: CGGTTTGACTTGGTATCCCTTT Reverse: CCGGAAGGACCGGATGTC

### Immunostaining

The cells were fixed with 3.7% formaldehyde for 10 minutes and then permeabilized and blocked with 5% FBS in PBST (0.1% Tween 20 in PBS) for 1 hour at room temperature. The cells were then stained with primary antibodies (anti-albumin, Abcam; anti-EpCAM, Abcam; anti-OC2, LSBio, Seattle, WA, USA; anti-cytokeratin 18, Sigma-Aldrich) for overnight, followed by staining with secondary antibodies for 2 hours at room temperature. DAPI (Sigma-Aldrich) was used for staining of cell nuclei. Image J was used for quantification.

### Western blotting

The MSC-secreted exosomes were lysed and quantified. Twenty micrograms of protein was separated on a 12% polyacrylamide gel and transferred to a PVDF membrane. The membrane was blocked by 5% BSA/TBST for 1 hour, and then probed with primary antibodies (CD9, Origene Technologies, Rockville, MD, USA; CD63, Novus Biologicals, Littleton, CO, USA) at 4 °C overnight. After TBST washing, the membrane was incubated with HRP-conjugated secondary antibodies at room temperature for 2 hours. After TBST washing, protein level was detected using SuperSignal™ West Femto Maximum Sensitivity Substrate (Thermo Fisher Scientific). Chemiluminescence imaging was captured by the UVP BioSpectrum 600 System (UVP LLC, Upland, CA, USA).

### Time-lapse imaging

The condition of the time-lapse imaging chamber (Sage Vision Co., Ltd, New Taipei City, Taiwan) was controlled using a temperature and humidity controller in an atmosphere containing 5% CO_2_ and 95% air. The culture dishes were placed in the chamber for time-lapse imaging.

### Hepatic differentiation

The MSC-CM-induced small oval cells are cultured in hepatic culture medium consisting of DMEM/F12 supplemented with 10% FBS and 1% PSG. Then, the differentiated cells were fixed and stained with PAS staining and immunostaining of cytokeratin 18.

### PAS staining

The cells were fixed with 3.7% formaldehyde for 10 minutes, and permeablized using 0.1% Triton X-100 for 10 minutes. Then, the cells were treated with 1% periodic acid for 5 minutes, and slightly washed with H_2_O. Finally, they were treated with Schiff’s fuchsin-sulfite solution (Sigma-Aldrich) for 1 hour, washed lightly with H_2_O, and dried.

### Statistical analysis

The data represented more than three independent experiments and was shown as the means ± standard deviation (SD). Student’s *t* tests were used for analysis of the significance of differences, and differences with *P* values of less than 0.05 were considered significant.

## Results

### MSC-CM treatment converted hepatocytes into small oval cells

**Fig. 1:**
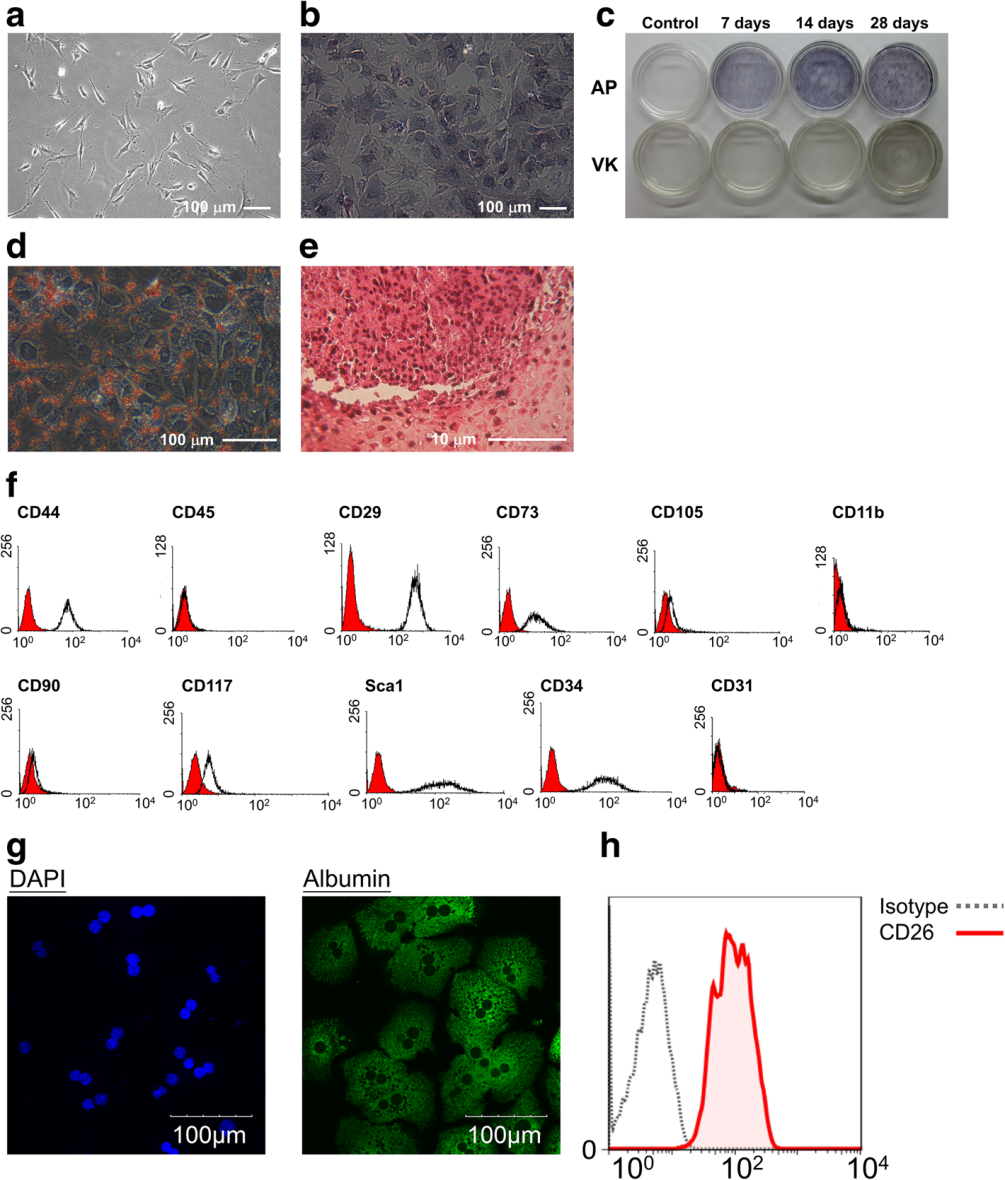
Characterization of murine MSCs and primary hepatocytes. MSCs were isolated from bone marrow. **a** Morphology of undifferentiated MSCs, and **b** MSCs under osteogenic differentiation with alkaline phosphatase staining on day 7. **c** Alkaline phosphatase and von Kossa staining of MSCs during osteogenic differentiation. **d** Oil red O staining of MSCs under adipogenic differentiation on day 14. **e** Safrinin O staining of MSCs under chondrogenic differentiation on day 21. **f** Surface marker staining of the MSCs by flow cytometry. **g** Albumin expression of in primary hepatocytes was detected by immunostaining. **h** CD26 staining of primary hepatocytes by flow cytometry

**Fig. 2:**
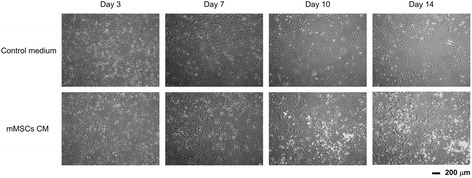
Paracrine signaling from MSC increased small oval cells in cultures of primary hepatocytes. The primary hepatocytes were treated with control medium and CM from MSCs for 14 days. *CM* conditioned medium, *mMSCs* murine mesenchymal stromal cells

Bone marrow-derived MSCs and primary hepatocytes were isolated from mice. The isolated MSCs showed a fibroblast-like morphology (Fig. 1a) and were able to be differentiated into bone, adipose tissue, and cartilage in vitro (Fig. 1b–e). The phenotypes of MSCs were characterized as positive for CD44, CD29, CD73, CD117, Sca1, and CD34; partially positive for CD105 and CD90; and negative for CD45, CD11b, and CD31 (Fig. 1f). Isolated primary hepatocytes expressed high levels of albumin (Fig. 1g). CD26, a marker of mature hepatocytes, was also highly expressed in the isolated primary hepatocytes (Fig. 1h). In order to investigate the paracrine effects of MSCs on hepatocytes, MSC-CM was collected to treat the primary hepatocytes. As shown in Fig. 2a, a few small, oval-shaped cells were observed from day 7 upon MSC-CM treatment. Subsequently, the number of small oval-shaped cells further increased on day 10 and day 14 in the MSC-CM treatment compared with control medium. In the control medium, most hepatocytes remained spread and flattened. The result showed that paracrine signaling of MSCs converted hepatocytes into small, oval-shaped cells.

### Confirmation of morphological changes under time-lapse microscopy

**Fig. 3:**
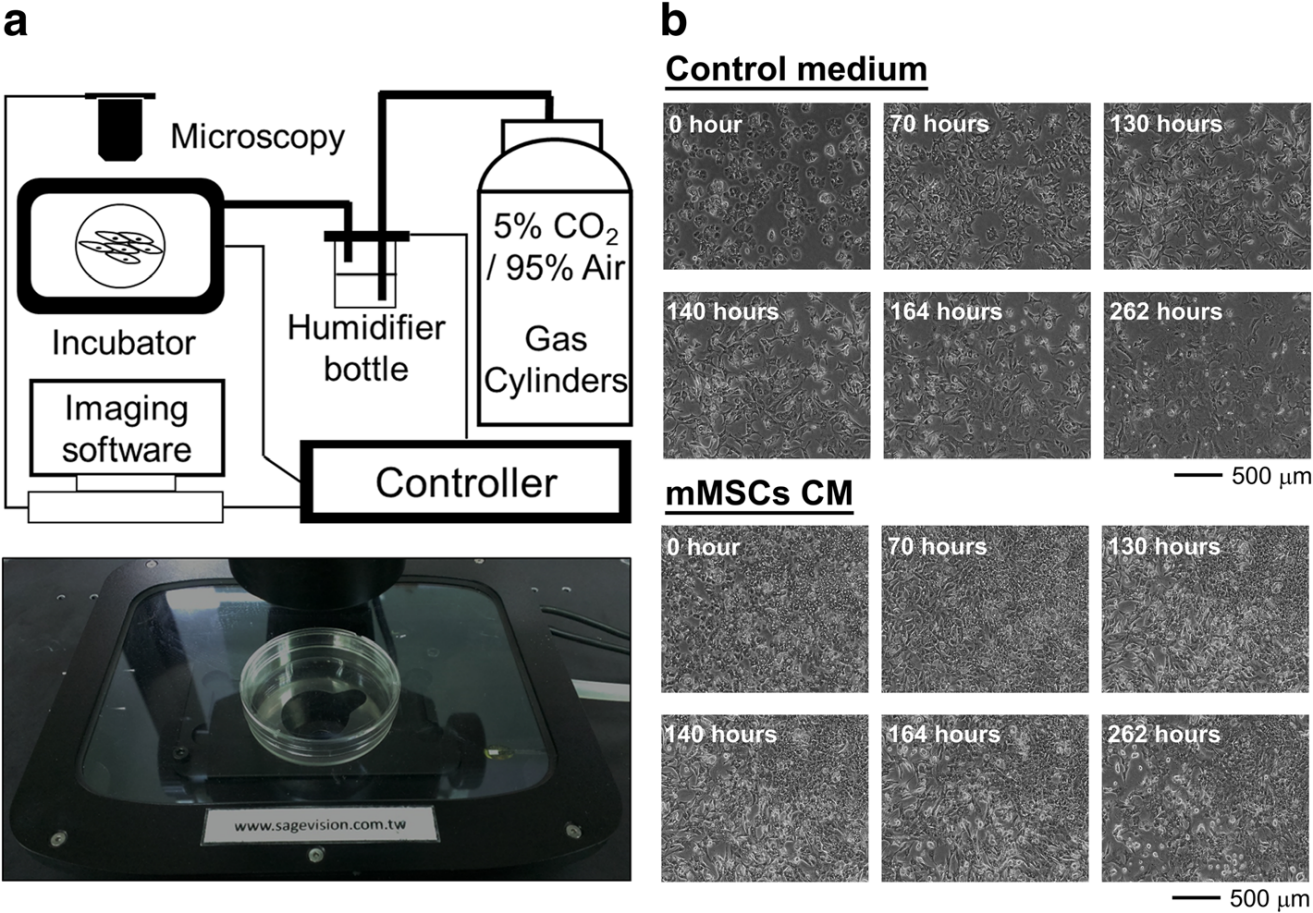
Paracrine signaling from MSCs induced morphological change in hepatocytes. **a** The instrument used for time-lapse imaging. **b** The time-lapse imaging of primary hepatocytes treated with control medium and CM from MSCs. *CM* conditioned medium, *mMSCs* murine mesenchymal stromal cells


In order to confirm the change of morphology induced by MSC-CM treatment, a time-lapse image system (Fig. 3a) was used for further observation. In the same cells, as shown in Fig. 3b and Additional files 1 and 2, hepatocytes changed their morphology from typical epithelial to fibroblast-like after 3 days of MSC-CM treatment. Subsequently, the cells further altered from fibroblast-like to small cells with oval shapes with continuous MSC-CM treatment for 3–11 days. In contrast, hepatocytes cultured in control medium maintained their flattened epithelial morphology (Fig. 3b and Additional files 1 and 2). The result confirmed the morphological change from typical epithelial in hepatocytes to small oval shape was induced by paracrine signaling of MSCs.

Additional file 1: mMSCs CM treatment 0~12 days. (WMV 59767 kb)



Additional file 2: Control medium treatment 0~12 days. (WMV 28392 kb)


### Changes of gene and surface marker expression induced by MSC-CM treatment

**Fig. 4:**
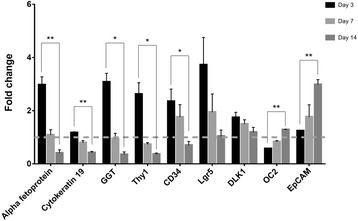
Paracrine signaling from MSCs upregulated mRNA expression of EpCAM and OC2 in hepatocytes. The mRNA expression level of alpha fetoprotein, cytokeratin 19, GGT, Thy1, CD34, Lgr5, DLK1, OC2, and EpCAM were analyzed in the hepatocytes by quantitative PCR. ^*^ *P* < 0.05; ^**^ *P* < 0.01

It has been known that when the liver is injured, hepatic oval cells as liver progenitor cells (LPCs) or transit-amplifying cells become abundant and highly express cytokeratin 19 and alpha fetoprotein [24–26]. In addition, Thy1, CD34, and γ-glutamyltranspeptidase (GGT) are reported as markers of hepatic oval cells [27]. Also, Lgr5 is expressed in LPCs in response to liver injury [28]. Therefore, the mRNA expression levels of oval cell markers, alpha fetoprotein, cytokeratin 19, GGT, Thy1, CD34, and Lgr5 were further analyzed in hepatocytes following MSC-CM treatment. As shown in Fig. 4, the mRNA expression level of alpha fetoprotein, cytokeratin 19, GGT, Thy1, CD34, and Lgr5 was upregulated on day 3, but downregulated on day 14 following treatment with CM from MSCs compared with control medium. In contrast, mRNA expression level of EpCAM, marker of intrahepatic stem cells and hepatoblasts, in hepatocytes was apparently upregulated upon 14 days of MSC-CM treatment. DLK1, a hepatoblast marker [29], was upregulated in hepatocytes with MSC-CM treatment. Onecut 2 (OC2) is reported to express in hepatic oval cells and is involved in controlling hepatoblast migration in early liver development [30]. In Fig. 2b, OC2 expression was also upregulated after 14 days of MSC-CM treatment. Collectively, these results indicated that paracrine signaling of MSCs upregulated expression of hepatic progenitor markers in conjunction with morphological changes.

### MSC-CM-induced small oval cells expressed hepatic progenitor markers

**Fig. 5:**
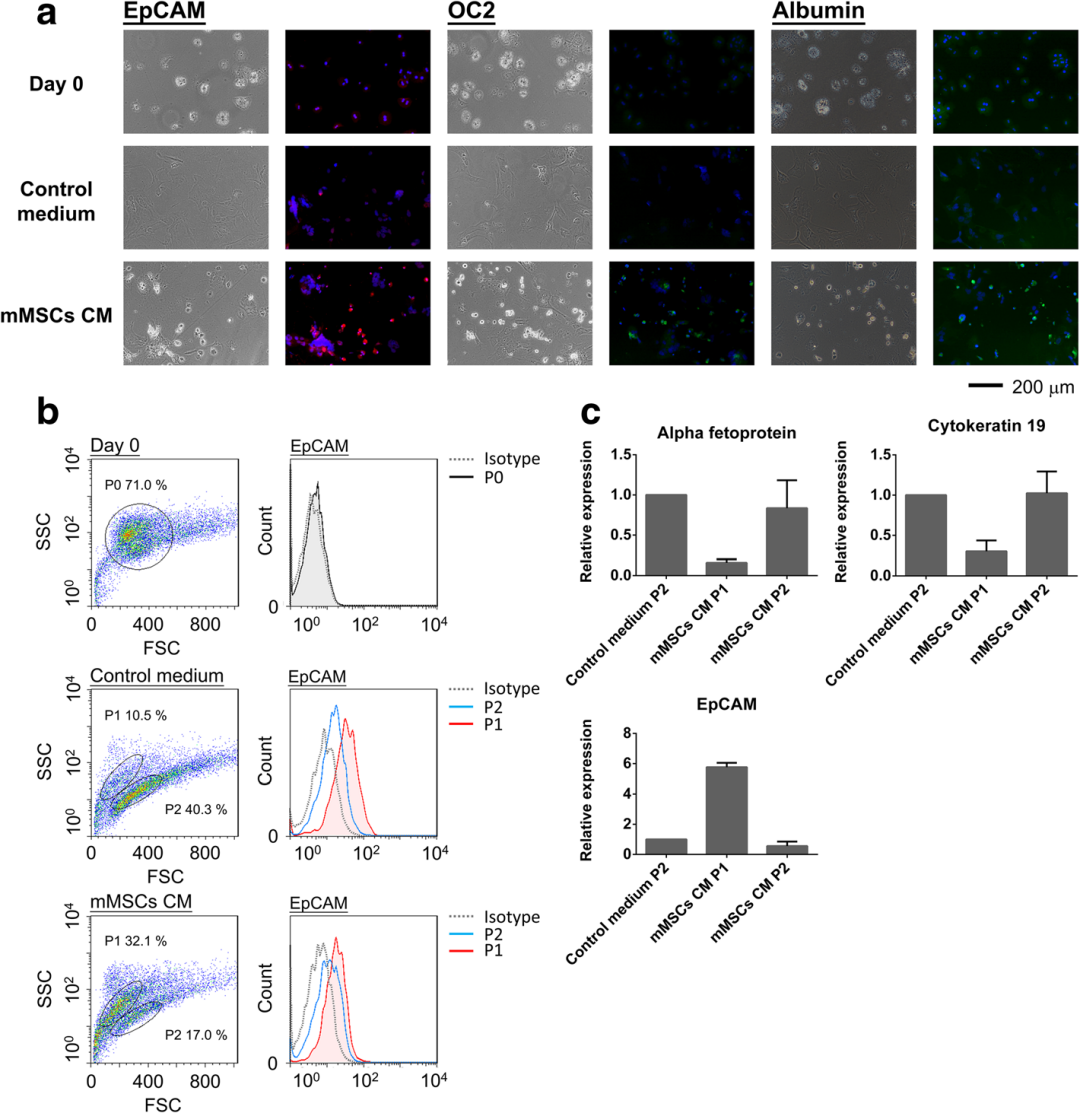
The MSC-CM-induced small oval cells expressed EpCAM and OC2. **a** Immunostaining of EpCAM, OC2, and albumin. **b** Quantitation of EpCAM-expressing cells by flow cytometry showed P1 (EpCAM^high^) and P2 (EpCAM^low^) populations in the hepatocytes with treatment of MSC-CM and control medium. **c** The mRNA expression level of alpha fetoprotein, cytokeratin 19, and EpCAM in the P1 and P2 cells were analyzed by quantitative PCR. *CM* conditioned medium, *mMSCs* murine mesenchymal stromal cells

As shown in Figs. 2 and 4, small oval-shaped cells were increased, and mRNA expression level of hepatoblast markers, EpCAM and OC2, was upregulated after MSC-CM treatment. In order to investigate expression of hepatoblast markers, EpCAM and OC2, in the small oval cells, immunostaining was used and showed that EpCAM and OC2 were highly expressed in the small oval cells (Fig. 5a). Also, albumin expression was highly expressed in the small oval cells. These results indicated that these small oval cells induced by the paracrine effect of MSCs were similar to EpCAM^+^ hepatoblasts as previous described [31]. Furthermore, in order to quantify EpCAM^+^ cells induced by MSC-CM treatment, the cells were analyzed by flow cytometry. In Fig. 5b, two subpopulations of cells, P1 and P2, were identified under flow cytometry after treatment with MSC-CM. P1 cells were smaller cells with higher EpCAM expression, while P2 cells were bigger cells with lower EpCAM expression. P1 cells increased in frequency with MSC-CM treatment compared to control medium. The flow cytometry results also supported the induction of EpCAM^high^ cells by MSC-CM.

To further characterize the two subpopulations, P1 and P2 cells were then sorted and expression of alpha fetoprotein, cytokeratin 19, and EpCAM was measured. P1 cells showed higher expression of EpCAM and lower expression of alpha fetoprotein as well as cytokeratin 19 compared to P2 cells. Conversely, P2 cells possessed higher expression of alpha fetoprotein and cytokeratin 19, but with a lower expression of EpCAM.

### MSC-CM-induced small oval cells can maturate and become proliferative hepatocytes

**Fig. 6:**
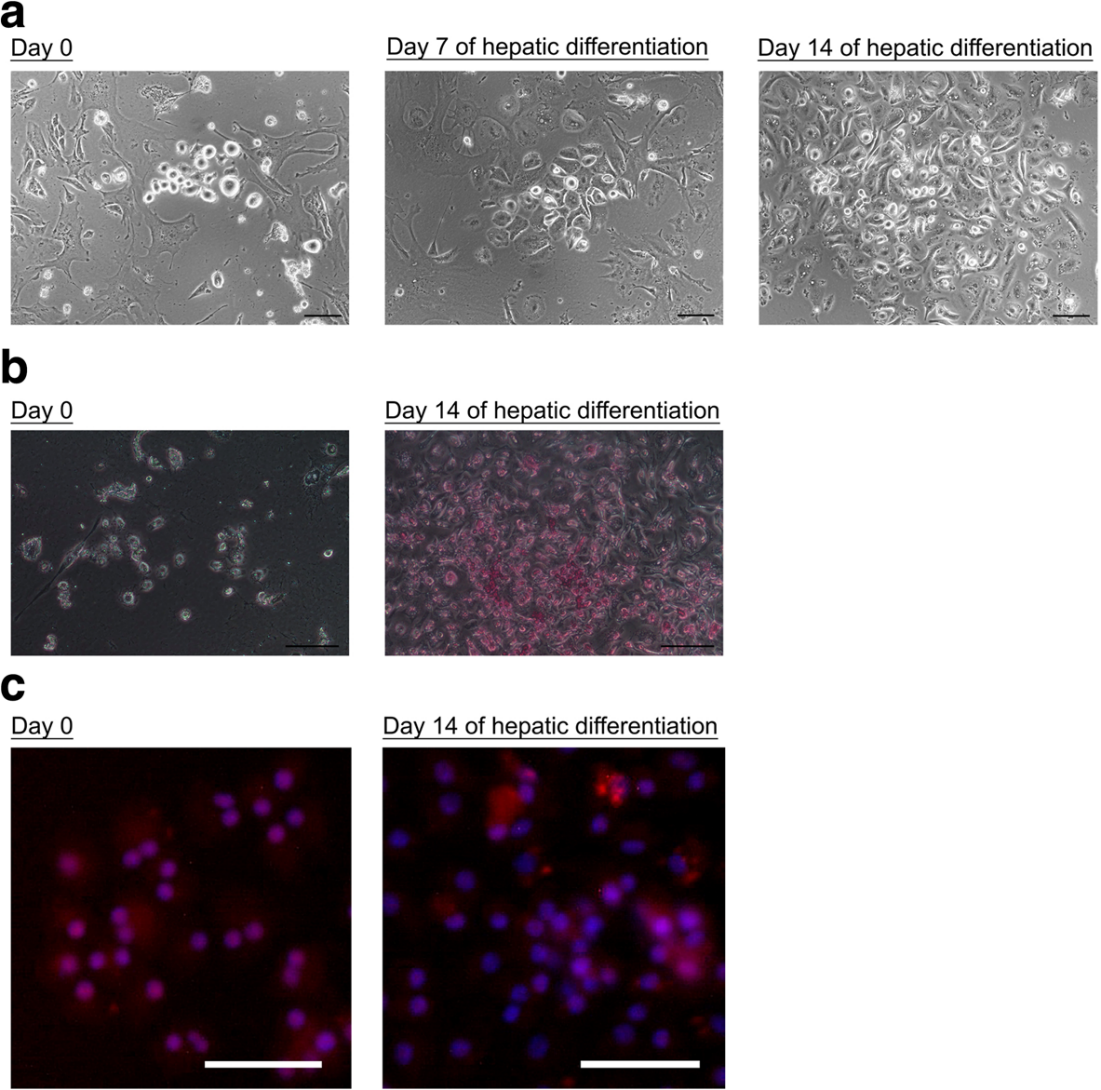
Hepatic differentiation of MSC-CM-induced small oval cells. **a** The MSC-CM-induced small oval cells were cultured in hepatic culture medium. **b** PAS staining and **c** immunostaining of cytokeratin 18 in MSC-CM-induced small oval cells at day 14 of hepatic differentiation. Scale bar: 100 μm

To investigate whether the MSC-CM-induced small oval cells can further maturate and differentiate to hepatocytes, these cells were moved to hepatic culture medium. In Fig. 6a, the small oval cells changed their morphology to epithelial cells and proliferated as a colony during hepatic differentiation. PAS staining showed glycogen storage during hepatic differentiation in the MSC-CM-induced small oval cells (Fig. 6b). The differentiated cells also expressed cytokeratin 18 as shown as Fig. 6c. The results indicated the MSC-CM-induced small oval cells could maturate and become proliferative hepatocytes.

### MSC-secreted exosomes converted mature hepatocytes to EpCAM^high^ small oval cells

**Fig. 7:**
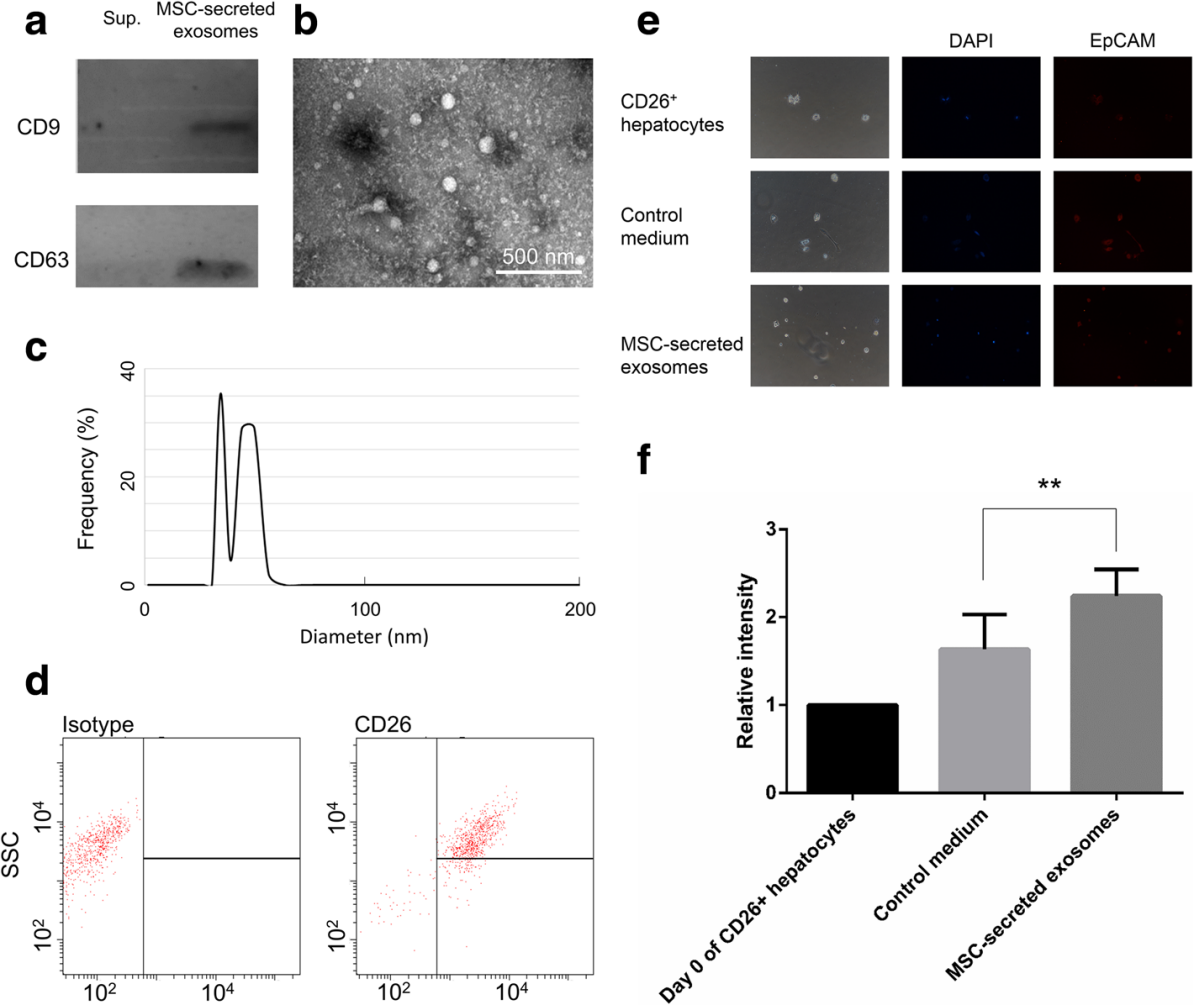
MSC-secreted exosomes induced EpCAM^high^ small oval cells from CD26^+^ hepatocytes. Characterization of MSC-secreted exosomes by **a** CD9 and CD63 expression, **b** dynamic light scattering analysis, and **c** TEM. **d** CD26^+^ hepatocytes were sorted and treated with control medium and 50 μg/ml MSC-secreted exosomes for 14 days. **e** The cells were stained with EpCAM immunostaining. **f** The normalization and quantification of EpCAM expression. ^*^ *P* < 0.05; ^**^ *P* < 0.01. Sup. Supernatant

In order to investigate whether MSC-secreted exosomes can induce the formation of small oval cells, MSC-CM was concentrated for further isolation of MSC-secreted exosomes. First, the exosomes were characterized by CD9 and CD63 expression (Fig. 7a). Also, by dynamic light scattering analysis and TEM, the diameter of exosomes were confirmed to be less to 100 μm (Fig. 7b-c). CD26^+^ mature hepatocytes (Fig. 7d) were sorted and then treated with MSC-secreted exosomes for 14 days. In Fig. 7e-f, it was shown that CD26^+^ hepatocytes were converted to small oval cells with higher EpCAM expression. The result indicated that the secreted exosomes in MSC-CM were important messengers to induce EpCAM^high^ small oval cells.

## Discussion

MSC transplantation exerts therapeutic effects in liver disease models, including acute and chronic liver failure. Additionally, MSC transplantation has been shown to improve the liver microenvironment, mediating restoration of liver function, reduction of inflammation, and augmentation of liver regeneration [32]. In our study, paracrine signaling from MSCs can induce the formation of hepatic oval cells via secretary exosomes. These findings could clarify the involvement of MSCs in therapies for liver diseases.

During liver regeneration, it is reported that the expression of neonatal markers, alpha fetoprotein and EpCAM, is upregulated [18, 33]. EpCAM is a marker of intrahepatic stem cells which present in normal and injured liver [18]. In addition, EpCAM is reported to express in undifferentiated stem cells and progenitors in development of organs, and drop to a very low level with terminal differentiation [34, 35]. During liver development of mice, EpCAM was expressed in liver buds as early as E9.5, decreased to low-level expression in hepatoblasts of E13.5, and not expressed when cells are switched onto specification to the hepatic fate [36]. The dynamic expression of EpCAM is also considered to correlate with plasticity of hepatocyte [37]. In addition, alpha fetoprotein and cytokeratin 19 are highly expressed in hepatic oval cells, which proliferate in the early stage of liver regeneration [25, 38]. Collectively, the above evidence supports the conversion of hepatocytes into hepatic progenitor cells.

Exosomes play an important role in cell-to-cell communication [9, 39]. By the transport system of exosomes, miRNAs are packaged and trafficked to recipient cells [40]. Moreover, many reports indicate that exosomal miRNAs can manipulate gene expression, and stimulate angiogenesis and migration in neighboring cells [41–43]. These secreted miRNAs can function as endogenous miRNAs to affect translation and manipulate cell signaling in recipient cells. Variety of miRNAs in exosomes are recorded in the ExoCarta database [44]. Although we found that MSC-secreted exosomes could induce formation of hepatic oval cells, further studies are needed to investigate which candidates of MSC exosomal miRNAs are involved in the formation of hepatic oval cells. In our study, not all of the hepatocytes were altered to EpCAM^high^ small oval cells with MSC-CM treatment, which may imply efficiency of exosome engulfing in hepatocytes and other important unknown key factors secreted from MSCs.

MSC-secreted growth factors and cytokines [4, 7] may also be involved in the process of liver regeneration. Hepatocyte growth factor (HGF) is an important growth factor to participate in liver regeneration [45]. In the early stage of liver regeneration, HGF expression is rapidly elevated from 3 to 48 hours and acts as an initiator of liver regeneration [46]. Also, genetic deletion of c-Met blocks liver regeneration and impairs liver functions, indicating that HGF/c-Met signaling is essential for liver regeneration [47, 48]. In addition, proteomics analysis also shows abundant HGF in MSC-derived exosomes [49]. Therefore, further investigation is required on whether HGF/c-Met signaling is involved in hepatocyte dedifferentiation.

In the studies, CD26^+^ hepatocytes were converted to small oval cells with higher EpCAM expression after treatment of concentrated MSC-secreted exosomes. Although the phenomenon was observed in the MSC-CM treatment, other factors which were not packaged into the exosomes could also be involved in the conversion of CD26^+^ hepatocytes to small oval cells with higher EpCAM expression. Also, another study analyzed the difference and intersection of the proteomic profile between MSC-CM and MSC-derived exosomes [49]. Therefore, the effectiveness of inducing hepatocyte dedifferentiation will need to be compared and investigated among MSC-CM, MSC-derived exosomes, and exosome-free MSC-CM. Furthermore, the key factors and regulators also need to be further elucidated.

By lineage tracing, hepatocytes can dedifferentiate to hepatic progenitor cells and then replenish hepatocytes in liver regeneration [20]. Also, there is some evidence indicating the increase of alpha fetoprotein expression, a marker of hepatic oval cells, after MSC transplantation in animal models [50, 51]. However, it is still unclear whether MSC transplantation can induce or increase the formation of hepatic oval cells in vivo. In the study, we demonstrated that paracrine signaling from MSCs can induce the formation of hepatic oval cells in vitro, and further investigation will be performed in future studies on whether MSCs transplantation and administration of MSC-CM and MSC-derived exosomes can induce recipient hepatocytes into hepatic oval cells in vivo.

MSC transplantation is thought to be quite safe, and many clinical trials have been carried out within the last decade [52, 53]. However, there are some concerns regarding the potential for tumorigenesis owing to the development of chromosomal aberrations in cell culture [54–56]. Alternatively, stem cell-derived molecules in CM, including cytokines, growth factors, and exosomes, can provide a more safe treatment method without cell transplantation. Therefore, analysis of the paracrine effects of MSCs may provide more feasible treatment guidelines in clinical applications.

## Conclusions

Paracrine signaling from MSCs induce the conversion of hepatocytes into progenitor oval cells via secretary exosomes. The finding provides evidence supporting the involvement of MSC-secreted exosomes during liver regeneration. Treatment with MSC-secreted exosomes may be an alternative approach to achieve therapeutic effects for liver diseases.

## Abbreviations

CM: Conditioned medium
EVs: Extracellular vesicles
HGF: Hepatocyte growth factor
LPCs: liver progenitor cells
MSCs: Mesenchymal stem cells
TEM: Transmission electron microscopy

## References

[1] English K, French A, Wood KJ (2010). Mesenchymal stromal cells: facilitators of successful transplantation?. Cell Stem Cell.

[2] Si YL, Zhao YL, Hao HJ, Fu XB, Han WD (2011). MSCs: Biological characteristics, clinical applications and their outstanding concerns. Ageing Res Rev.

[3] Kuo TK, Hung SP, Chuang CH, Chen CT, Shih YR, Fang SC (2008). Stem cell therapy for liver disease: parameters governing the success of using bone marrow mesenchymal stem cells. Gastroenterology.

[4] Christ B, Bruckner S, Winkler S (2015). The therapeutic promise of mesenchymal stem cells for liver restoration. Trends Mol Med.

[5] Stock P, Bruckner S, Winkler S, Dollinger MM, Christ B (2014). Human bone marrow mesenchymal stem cell-derived hepatocytes improve the mouse liver after acute acetaminophen intoxication by preventing progress of injury. Int J Mol Sci.

[6] Winkler S, Hempel M, Bruckner S, Mallek F, Weise A, Liehr T (2015). Mouse white adipose tissue-derived mesenchymal stem cells gain pericentral and periportal hepatocyte features after differentiation in vitro, which are preserved in vivo after hepatic transplantation. Acta Physiol (Oxf).

[7] Liang X, Ding Y, Zhang Y, Tse HF, Lian Q (2014). Paracrine mechanisms of mesenchymal stem cell-based therapy: current status and perspectives. Cell Transplant.

[8] van Poll D, Parekkadan B, Cho CH, Berthiaume F, Nahmias Y, Tilles AW (2008). Mesenchymal stem cell-derived molecules directly modulate hepatocellular death and regeneration in vitro and in vivo. Hepatology.

[9] Raposo G, Stoorvogel W (2013). Extracellular vesicles: exosomes, microvesicles, and friends. J Cell Biol.

[10] Akyurekli C, Le Y, Richardson RB, Fergusson D, Tay J, Allan DS (2015). A systematic review of preclinical studies on the therapeutic potential of mesenchymal stromal cell-derived microvesicles. Stem Cell Rev.

[11] Lin J, Li J, Huang B, Liu J, Chen X, Chen X-M (2015). Exosomes: novel biomarkers for clinical diagnosis. Sci World J..

[12] Han C, Sun X, Liu L, Jiang H, Shen Y, Xu X (2016). Exosomes and their therapeutic potentials of stem cells. Stem Cells Int..

[13] Herrera MB, Fonsato V, Gatti S, Deregibus MC, Sordi A, Cantarella D (2010). Human liver stem cell-derived microvesicles accelerate hepatic regeneration in hepatectomized rats. J Cell Mol Med.

[14] Li T, Yan Y, Wang B, Qian H, Zhang X, Shen L (2013). Exosomes derived from human umbilical cord mesenchymal stem cells alleviate liver fibrosis. Stem Cells Dev.

[15] Fausto N (2004). Liver regeneration and repair: hepatocytes, progenitor cells, and stem cells. Hepatology.

[16] Fausto N, Campbell JS (2003). The role of hepatocytes and oval cells in liver regeneration and repopulation. Mech Dev.

[17] Dolle L, Theise ND, Schmelzer E, Boulter L, Gires O, van Grunsven LA (2015). EpCAM and the biology of hepatic stem/progenitor cells. Am J Physiol Gastrointest Liver Physiol.

[18] Okabe M, Tsukahara Y, Tanaka M, Suzuki K, Saito S, Kamiya Y (2009). Potential hepatic stem cells reside in EpCAM+ cells of normal and injured mouse liver. Development.

[19] Gires O (2012). EpCAM in hepatocytes and their progenitors. J Hepatol.

[20] Tarlow BD, Pelz C, Naugler WE, Wakefield L, Wilson EM, Finegold MJ (2014). Bipotential adult liver progenitors are derived from chronically injured mature hepatocytes. Cell Stem Cell.

[21] Jiang WC, Cheng YH, Yen MH, Chang Y, Yang VW, Lee OK (2014). Cryo-chemical decellularization of the whole liver for mesenchymal stem cells-based functional hepatic tissue engineering. Biomaterials.

[22] Klaunig JE, Goldblatt PJ, Hinton DE, Lipsky MM, Chacko J, Trump BF (1981). Mouse liver cell culture. I Hepatocyte isolation. In vitro..

[23] Klaunig JE, Goldblatt PJ, Hinton DE, Lipsky MM, Trump BF (1981). Mouse liver cell culture. II Primary culture. In vitro..

[24] Oh SH, Hatch HM, Petersen BE (2002). Hepatic oval ‘stem’ cell in liver regeneration. Semin Cell Dev Biol.

[25] Verhulst S, Best J, van Grunsven LA, Dolle L (2015). Advances in hepatic stem/progenitor cell biology. EXCLI J.

[26] Matthews VB, Yeoh GC (2005). Liver stem cells. IUBMB Life.

[27] Shin S, Kaestner KH (2014). The origin, biology, and therapeutic potential of facultative adult hepatic progenitor cells. Curr Top Dev Biol..

[28] Huch M, Dorrell C, Boj SF, van Es JH, Li VS, van de Wetering M (2013). In vitro expansion of single Lgr5+ liver stem cells induced by Wnt-driven regeneration. Nature.

[29] Tanimizu N, Nishikawa M, Saito H, Tsujimura T, Miyajima A (2003). Isolation of hepatoblasts based on the expression of Dlk/Pref-1. J Cell Sci.

[30] Margagliotti S, Clotman F, Pierreux CE, Beaudry JB, Jacquemin P, Rousseau GG (2007). The Onecut transcription factors HNF-6/OC-1 and OC-2 regulate early liver expansion by controlling hepatoblast migration. Dev Biol.

[31] Tanaka M, Okabe M, Suzuki K, Kamiya Y, Tsukahara Y, Saito S (2009). Mouse hepatoblasts at distinct developmental stages are characterized by expression of EpCAM and DLK1: drastic change of EpCAM expression during liver development. Mech Dev.

[32] Zhang Z, Wang FS (2013). Stem cell therapies for liver failure and cirrhosis. J Hepatol.

[33] Petropoulos C, Andrews G, Tamaoki T, Fausto N (1983). alpha-Fetoprotein and albumin mRNA levels in liver regeneration and carcinogenesis. J Biol Chem.

[34] Trzpis M, McLaughlin PM, de Leij LM, Harmsen MC (2007). Epithelial cell adhesion molecule: more than a carcinoma marker and adhesion molecule. Am J Pathol.

[35] de Boer CJ, van Krieken JH, Janssen-van Rhijn CM, Litvinov SV (1999). Expression of Ep-CAM in normal, regenerating, metaplastic, and neoplastic liver. J Pathol.

[36] Miyajima A, Tanaka M, Itoh T (2014). Stem/progenitor cells in liver development, homeostasis, regeneration, and reprogramming. Cell Stem Cell.

[37] Huch M, Dolle L (2016). The plastic cellular states of liver cells: are EpCAM and Lgr5 fit for purpose?. Hepatology.

[38] Kuhlmann WD, Peschke P (2006). Hepatic progenitor cells, stem cells, and AFP expression in models of liver injury. Int J Exp Pathol.

[39] EL Andaloussi S, Mager I, Breakefield XO, Wood MJ (2013). Extracellular vesicles: biology and emerging therapeutic opportunities. Nat Rev Drug Discov.

[40] Zhang J, Li S, Li L, Li M, Guo C, Yao J (2015). Exosome and exosomal microRNA: trafficking, sorting, and function. Genomics Proteomics Bioinformatics.

[41] Zhou W, Fong MY, Min Y, Somlo G, Liu L, Palomares MR (2014). Cancer-secreted miR-105 destroys vascular endothelial barriers to promote metastasis. Cancer Cell.

[42] Umezu T, Ohyashiki K, Kuroda M, Ohyashiki JH (2013). Leukemia cell to endothelial cell communication via exosomal miRNAs. Oncogene.

[43] van Balkom BW, de Jong OG, Smits M, Brummelman J, den Ouden K, de Bree PM (2013). Endothelial cells require miR-214 to secrete exosomes that suppress senescence and induce angiogenesis in human and mouse endothelial cells. Blood.

[44] Simpson RJ, Kalra H, Mathivanan S. ExoCarta as a resource for exosomal research. J Extracell Vesicles. 2012;1. doi:10.3402/jev.v1i0.18374 10.3402/jev.v1i0.18374 PMC3760644 24009883

[45] Michalopoulos GK (2007). Liver regeneration. J Cell Physiol.

[46] Pediaditakis P, Lopez-Talavera JC, Petersen B, Monga SP, Michalopoulos GK (2001). The processing and utilization of hepatocyte growth factor/scatter factor following partial hepatectomy in the rat. Hepatology.

[47] Factor VM, Seo D, Ishikawa T, Kaposi-Novak P, Marquardt JU, Andersen JB et al. Loss of c-Met disrupts gene expression program required for G2/M progression during liver regeneration in mice. PloS One. 2010;5(9). doi:10.1371/journal.pone.0012739 10.1371/journal.pone.0012739 PMC2940888 20862286

[48] Ishikawa T, Factor VM, Marquardt JU, Raggi C, Seo D, Kitade M (2012). Hepatocyte growth factor/c-met signaling is required for stem-cell-mediated liver regeneration in mice. Hepatology.

[49] Lai RC, Tan SS, Teh BJ, Sze SK, Arslan F, de Kleijn DP (2012). Proteolytic potential of the MSC exosome proteome: implications for an exosome-mediated delivery of therapeutic proteasome. Int J Proteomics..

[50] Cai Y, Zou Z, Liu L, Chen S, Chen Y, Lin Z (2015). Bone marrow-derived mesenchymal stem cells inhibits hepatocyte apoptosis after acute liver injury. Int J Clin Exp Pathol.

[51] Wang M, Zhang X, Xiong XI, Yang Z, Li P, Wang J (2016). Bone marrow mesenchymal stem cells reverse liver damage in a carbon tetrachloride-induced mouse model of chronic liver injury. In Vivo.

[52] Wei X, Yang X, Han ZP, Qu FF, Shao L, Shi YF (2013). Mesenchymal stem cells: a new trend for cell therapy. Acta Pharmacol Sin.

[53] Keating A (2012). Mesenchymal stromal cells: new directions. Cell Stem Cell.

[54] Barkholt L, Flory E, Jekerle V, Lucas-Samuel S, Ahnert P, Bisset L (2013). Risk of tumorigenicity in mesenchymal stromal cell-based therapies--bridging scientific observations and regulatory viewpoints. Cytotherapy.

[55] Grigorian AS, Kruglyakov PV, Taminkina UA, Efimova OA, Pendina AA, Voskresenskaya AV (2010). Alterations of cytological and karyological profile of human mesenchymal stem cells during in vitro culturing. Bull Exp Biol Med.

[56] Ben-David U, Mayshar Y, Benvenisty N (2011). Large-scale analysis reveals acquisition of lineage-specific chromosomal aberrations in human adult stem cells. Cell Stem Cell.

